# The Oldest of Old Male C57B/6J Mice Are Protected from Sarcopenic Obesity: The Possible Role of Skeletal Muscle Protein Kinase B Expression

**DOI:** 10.3390/ijms251910278

**Published:** 2024-09-24

**Authors:** Thomas H. Reynolds, Noa Mills, Dakembay Hoyte, Katy Ehnstrom, Alex Arata

**Affiliations:** Health and Human Physiological Sciences Department, Skidmore College, Saratoga Springs, NY 12866, USA

**Keywords:** aging, body composition, glucose metabolism, skeletal muscle, protein kinase B

## Abstract

The impact of aging on body composition and glucose metabolism is not well established in C57BL/6J mice, despite being a common pre-clinical model for aging and metabolic research. The purpose of this study was to examine the effect of advancing age on body composition, in vivo glucose metabolism, and skeletal muscle AKT expression in young (Y: 4 months old, *n* = 7), old (O: 17–18 months old, *n* = 10), and very old (VO: 26–27 month old, *n* = 9) male C57BL/6J mice. Body composition analysis, assessed by nuclear magnetic resonance, demonstrated O mice had a significantly greater fat mass and body fat percentage when compared to Y and VO mice. Furthermore, VO mice had a significantly greater lean body mass than both O and Y mice. We also found that the VO mice had greater AKT protein levels in skeletal muscle compared to O mice, an observation that explains a portion of the increased lean body mass in VO mice. During glucose tolerance (GT) testing, blood glucose values were significantly lower in the VO mice when compared to the Y and O mice. No age-related differences were observed in insulin tolerance (IT). We also assessed the glucose response to AMPK activation by 5-aminoimidazole-4-carboxamide-1-β-D-ribofuranoside (AICAR). The change in blood glucose following AICAR administration was significantly reduced in VO mice compared to Y and AG mice. Our findings indicate that lean body mass and AKT2 protein expression in muscle are significantly increased in VO mice compared to O mice. The increase in AKT2 likely plays a role in the greater lean body mass observed in the oldest of old mice. Finally, despite the increased GT, VO mice appear to be resistant to AMPK-mediated glucose uptake.

## 1. Introduction

Approximately 30% of the world’s population is obese [1]. From 1975 to 2016, the international rate of obesity increased six-fold to 671 million [2,3], a rate that is expected to increase by 50% in the next decade [1]. The public health implications of the increasing obesity prevalence are particularly concerning because obesity is highly associated with type 2 diabetes and heart disease. Furthermore, aging appears to exasperate obesity; in the United States the age-adjusted prevalence of severe obesity is greater in adults aged 40–59 compared to adults aged 20–39 [4]. This is particularly concerning since the number of individuals between 45 and 64 years of age is expected to increase by close to 20% by 2060 [5].

The combination of aging and obesity results in a loss of a lean body mass and muscle function [6], leading to sarcopenic obesity [7]. In fact, obesity may increase susceptibility to age-related reductions in muscle size and function by increasing pro-inflammatory cytokines, altering sex hormone levels and action, reducing type II fast-twitch muscle fiber expression, and by the lipotoxicity of fat accumulation within muscle [7]. Sarcopenic obesity has devastating consequences beyond muscle function and mobility, such as cardiometabolic disease and increases in all-cause mortality [8,9]. The cause of sarcopenic obesity is clearly multi-factorial and mechanistic clinical studies are difficult due to the gradual changes in body composition over decades. To learn more about the mechanisms responsible for obesity and aging pre-clinical mouse models have been used extensively. The C56BL/6 mouse strain is the most commonly employed laboratory mouse for the study of obesity and aging. According to the National Library of Medicine’s PubMed.gov search portal, over the last ten years close to 7000 studies have examined obesity or aging in C57BL/6 mice (https://pubmed.ncbi.nlm.nih.gov, accessed on 12 June 2024), but only a few studies have investigated sarcopenic obesity in aged mice [10,11,12]. Perhaps pre-clinical studies of aging mice can provide a model allowing for insights into the mechanisms responsible for sarcopenic obesity.

Protein kinase B, also known as AKT, is a serine/threonine kinase that regulates cellular growth, metabolism, apoptosis, and differentiation [13]. AKT controls skeletal muscle size by stimulating protein synthesis and suppressing protein degradation [14,15]. The role AKT plays in age-related muscle atrophy is not clear, as some reports show that AKT expression and signaling are not altered or increased with advancing age [16,17]. More recently, it has been shown that the mammalian target of rapamycin (mTOR), a purported downstream target of AKT that controls mRNA translation, is chronically activated in age-related muscle atrophy [15,18]. Alternatively, overload-induced hypertrophy and recovery from atrophy are both impaired in aged rats, a process that appears to be related to changes in AKT signaling [19]. The impact of aging and obesity on AKT expression and signaling has not been evaluated, despite AKT’s dual role in insulin action and the regulation of muscle size. Perhaps AKT may play a role in the development of sarcopenic obesity.

The purpose of the present study was to examine the impact of aging on body composition, in vivo glucose metabolism, AKT expression, and energy homeostasis in young (Y), old (O), and very old (VO) mice. To accomplish this, we studied male C57BL/6J mice at 4 months, 17–18 months, and 26–27 months old. Our findings reveal that AKT expression and lean body mass are increased in VO compared to O mice. We also demonstrate that changes in body composition, glucose metabolism, and energy homeostasis do not change linearly with advancing age, as the VO mice appeared to reverse many of the age-related changes observed in the O mice.

## 2. Results

### 2.1. Body Composition

To assess body composition, we used NMR (Bruker, LF-50 BCA, Billerica, MA, USA), a method that is highly correlated to the chemical analysis of body composition in mice [20]. As shown in Table 1, when compared to Y mice, O mice had significantly greater body mass, fat mass, and % body fat, without any significant differences in lean body mass. Although body mass was significantly higher in VO mice, this appears to be due to significantly greater lean body mass as fat mass was significantly lower when compared to O mice. Fat mass and body fat percentage were not significantly different in VO mice compared to Y mice. These results indicate that body composition does not change in a linear fashion with advancing age in mice.

### 2.2. GT Testing

**Figure 1:**
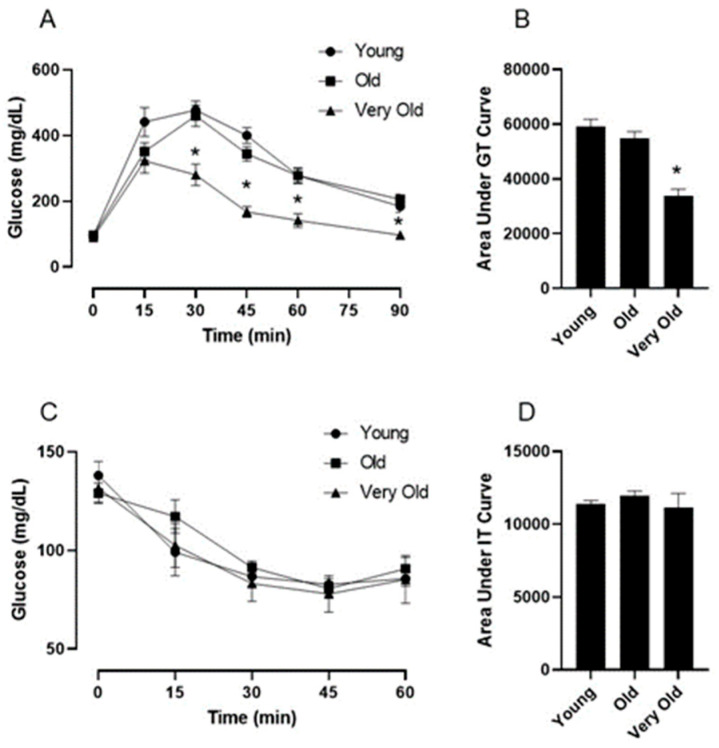
Advancing age improves glucose tolerance without impacting insulin tolerance in male C56BL/6J mice. Panel (**A**) shows the blood glucose response to an intraperitoneal injection of glucose (2 g/kg). Panel (**B**) is the area under the glucose tolerance curve. Panel (**C**) shows the blood glucose response to an intraperitoneal injection of insulin (0.75 U/kg). Panel (**D**) is the area under the insulin tolerance curve. Following a significant one-way repeated measures ANOVA, the Tukey post hoc test was used to locate statistically significant differences. * Significantly different from all other groups by Tukey post hoc test. N = 7–10 mice per group.

To assess glucose tolerance, mice were fasted overnight and then injected intraperitoneally with glucose (2 mg/g body weight). Glucose levels in tail vein blood samples were assessed at baseline and every 15 min for 90 min following the glucose injection. Fasted basal blood glucose values were not significantly different among the Y, O, and VO mice (Figure 1A). Surprisingly, the blood glucose values in the VO mice were significantly lower during the GT test when compared to both the Y and O mice (Figure 1A,B); however, no differences existed between the Y and O mice.

### 2.3. IT Testing

To determine if the age-related differences in GT were related to insulin sensitivity, we conducted an IT tolerance test. Mice were fasted for six hours and then injected intraperitoneally with insulin (0.75 U/kg). Glucose levels in tail vein blood samples were assessed at baseline and every 15 min for 90 min following the insulin injection. As shown in Figure 1C,D, insulin significantly reduced blood glucose in all mice, but there were no age-related differences. This observation indicates that changes in insulin sensitivity do not explain the lower glucose values during the GT test in VO mice.

### 2.4. AT Testing

**Figure 2:**
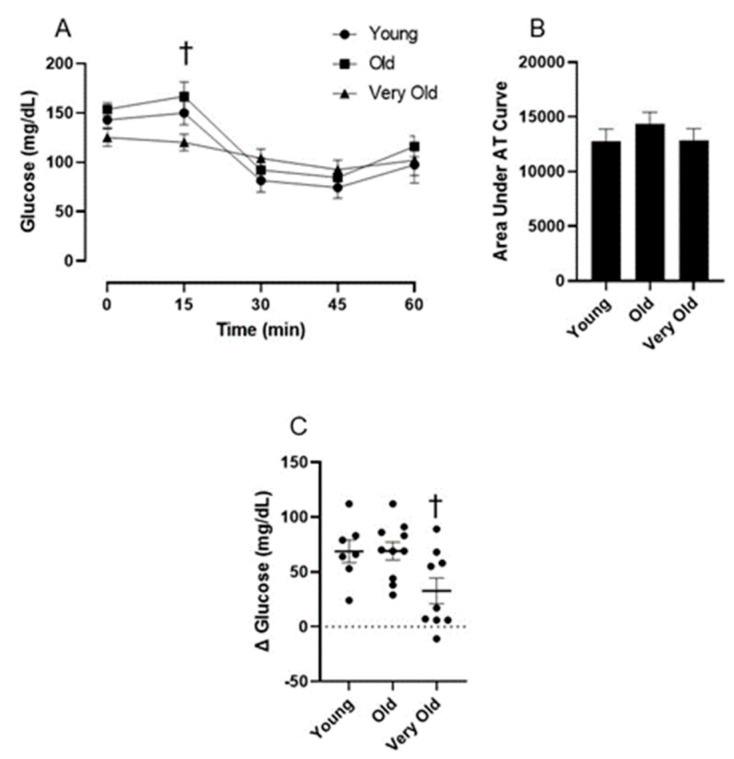
Advancing age reduces AICAR’s ability to lower blood glucose in male C56BL/6J mice. Panel (**A**) shows the blood glucose response to an intraperitoneal injection of AICAR (250 mg/kg). Panel (**B**) is the area under the AICAR tolerance curve. Panel (**C**) shows the relative change in blood glucose from basal to 45 min following the AICAR injection. Following a significant one-way repeated measures ANOVA, the Tukey post hoc test was used to locate statistically significant differences. † Significantly different from O group by Tukey post hoc test. N = 7–10 mice per group.

The impact of advancing age on the glucose response to AMPK activation is not known. Therefore, we administered the AMPK activator, AICAR, to mice following a six-hour fast and assessed blood glucose at baseline and 15, 30, 45, and 60 min following the AICAR injection. As shown in Figure 2A, AICAR resulted in a significant reduction in blood glucose from basal (time main effect: *p* = 0.0001) and it appears that age influences the blood glucose response to AICAR over time (age × time interaction: *p* = 0.0023). Furthermore, the relative change in glucose from basal to 45 min following the AICAR injection was significantly reduced in the VO mice compared to the O mice (Figure 2C).

### 2.5. Energy Homeostasis

**Figure 3:**
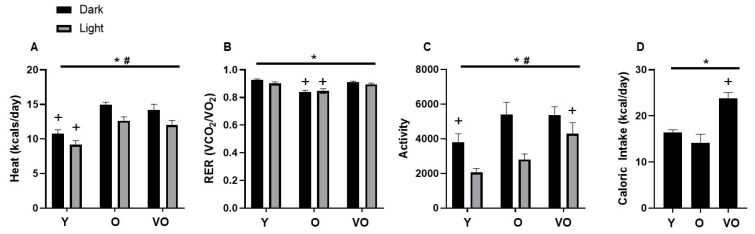
The effect of advancing age on energy homeostasis in male C56BL/6J mice. Panel (**A**): heat; Panel (**B**): RER; Panel (**C**): activity; Panel (**D**): caloric intake. Following a significant 2 × 2 (age × light phase) ANOVA, the Tukey post hoc test was used to locate statistically significant differences. * Significant age effect, # significant lighting phase effect, + significantly different from all other groups with similar light phase by Tukey post hoc test. N = 7–10 mice per group.

Energy expenditure was assessed by indirect calorimetry using a metabolic cage system. As shown in Figure 3A, energy expenditure was higher in O and VO mice compared to Y mice; however, this age effect appears to be dependent on body composition as these differences are not observed when the data are expressed relative to body weight or lean body mass. Figure 3B shows that RER is lower in O mice compared to Y and VO mice, indicating difference in substrate utilization. No differences were observed in RER between Y and VO mice. In the dark phase, total physical activity was significantly higher in O and VO mice compared to Y mice; however, in the light phase, physical activity was higher in the VO mice compared to the Y and O mice (Figure 3C). Figure 3D shows that caloric intake was significantly greater in the VO mice compared to the Y and O mice.

### 2.6. AKT Protein Expression

**Figure 4:**
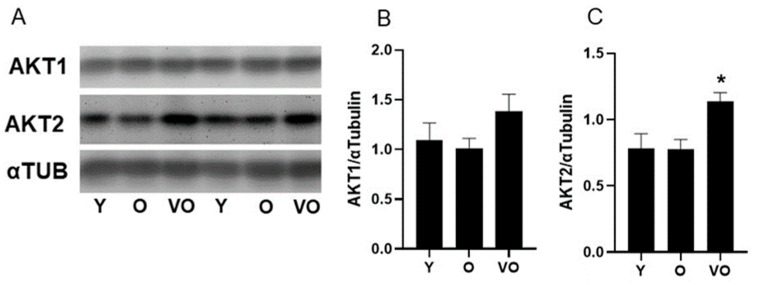
The effect of advancing age on AKT protein expression in male C56BL/6J mice. Panel (**A**): representative immunoblots for AKT1, AKT2, and α-tubulin; Panel (**B**): AKT1; Panel (**C**): AKT2; following a significant one-way ANOVA, the Tukey post hoc test was used to locate statistically significant differences. * Significantly different from all other groups by Tukey post hoc test. N = 6–8 mice per group.

To gain insight into potential mechanisms that control lean body mass, we assessed the levels of AKT1 and AKT2 in skeletal muscle from Y, O, and VO mice. As shown in Figure 4, there is no change in AKT1 and AKT2 levels between Y and O mice, indicating that aging up to 18 months does not alter AKT protein expression. However, AKT1 and AKT2 are higher in muscles from VO mice compared to Y and O mice, and this increase is statistically significant for AKT2 expression (Figure 4A,C). A significant positive relationship between AKT2 and lean body mass existed (R = 0.50, *p* = 0.04) and a trend for a relationship between AKT1 and lean body mass was also evident (R = 0.43, *p* = 0.09).

## 3. Discussion

The purpose of the present study was to examine the impact of aging on body composition, in vivo glucose metabolism, AKT expression, and energy homeostasis in Y, O, and VO C57BL/6J mice. The most interesting observation is that the VO mice are resistant to sarcopenic obesity as the VO mice had substantially less adipose tissue and greater lean tissue than O mice. These changes in body composition in the oldest of old mice were accompanied by an increase in AKT protein expression. A strong positive relationship existed between skeletal muscle AKT2 levels and lean body mass. Despite the increase in AKT protein expression in VO mice, IT was similar among the Y, O, and VO mice. GT was greater in VO mice compared to Y and O mice; however, VO mice were resistant to AICAR-stimulated glucose metabolism, implicating age-related changes in AMPK’s ability to lower blood glucose.

The present study demonstrates that lean body mass was higher and fat mass was lower in the VO mice compared to the O mice, indicating a reversal of sarcopenic obesity in the oldest of old mice. Surprisingly, lean body mass was also higher in VO mice when compared to Y mice and fat mass was quite similar. These results are limited to total lean body mass as our MRI analysis cannot decipher between muscle and bone tissue. Nonetheless, advancing age results in a loss of lean tissue, namely skeletal muscle and bones mass in both humans [21,22] and C57BL/6J mice [23,24,25]. Our findings are difficult to explain but confirm previous work showing a significantly higher lean body mass in 28-month-old male C57BL/6J mice compared to 20-month-old mice [26].

One potential explanation for the increased lean body mass in VO mice is skeletal muscle AKT expression, as the kinase plays a critical role in regulating both protein synthesis and degradation [15]. We observed a significant increase in AKT2 and a trend for an increase in AKT1 in skeletal muscle from VO mice compared to O mice. Our findings are strikingly similar to the observation that the overexpression of the AKT1 isoform increased muscle fiber size and reduced dietary-induced obesity [27], albeit our increases in AKT2 were more robust than the increases in AKT1. Perhaps the increase in AKT expression observed in the present study is a compensatory response to the loss of muscle mass in the O mice. This idea is supported by evidence that shows AKT2 expression and downstream signaling are increased in response to denervation-induced muscle atrophy [28] as are the AKT-regulated atrogenes MURF1 and MAFbx [29]. In other words, the increase in AKT expression observed presently in VO mice is likely an adaptive response that over-compensated for the loss of muscle mass that occurred earlier in the aging process. Therefore, it is possible that the present increase in AKT protein levels in the VO mice allowed for the recovery of lean tissue. This idea should be taken with caution as we did not directly assess muscle atrophy but assessed lean body mass via magnetic resonance, although skeletal muscle is the largest component of lean body mass.

We observed dramatic differences in body composition among the Y, O, and VO mice. The VO mice weighed less and possessed substantially less fat mass compared to the O mice. At first glance, the dramatically lower body weight and fat mass in the VO mice suggests severe wasting, but the loss of fat occurred in the face of increased lean body mass resulting in a body composition that was similar to Y mice. In an attempt to explain the improvement in body composition in the VO mice, we assessed energy homeostasis by measuring energy expenditure, physical activity, and caloric intake. Changes in energy expenditure do not appear to explain the changes in fat mass when expressed as kcal/day. For example, O mice had higher energy expenditure, but greater fat mass compared to Y mice, but VO mice had substantially less fat mass and no difference in energy expenditure compared to O mice. However, when energy expenditure data are normalized to lean body mass or body weight, significant differences emerge for O mice compared to the Y and VO mice, illustrating the challenges of interpreting metabolic cage data in animals with different body composition. Stern et al. [30] demonstrated higher energy expenditure in 15-month-old mice but not in 27-month-old mice compared to young mice when expressed kJ/mouse; findings that did not persist when energy expenditure was normalized to either body weight or lean body mass.

Unlike energy expenditure, respiratory exchange ratio (RER) provides an indication of substrate utilization that is independent of lean body mass or body weight. Interestingly, we observed a significantly lower RER in O mice when compared to Y and VO mice indicating age-dependent changes in energy substrate utilization, a finding quite similar to Stern et al. [30]. Increased activity in the light phase as well as increased caloric intake might explain not only the higher RER, but also the higher energy expenditure of our VO mice compared to the O and Y mice, respectively. It is surprising that we observed higher levels of physical activity and caloric intake in the VO mice, a finding that would be expected to promote a leaner phenotype, particularly if the increased physical activity exceeds the increase in caloric intake over a prolonged time period. The lower RER in O compared to Y mice does not seem to be associated with activity levels or caloric intake, but could possibly be related to greater lipid availability due to the increased fat mass of the O mice.

Information regarding changes in glucose homeostasis across the life span of C57BL/6J mice is limited and results from existing studies are variable. The present study demonstrates that 26–27-month-old male C57BL/6J mice had significantly lower glucose values during an intraperitoneal GT test. The improved GT in VO mice does not appear to be due to insulin resistance, as IT testing revealed no differences among VO, O, and Y mice. Surprisingly, our previous work examining the impact of age and sex on glucose homeostasis showed that 18-month-old male C57BL/6J mice had a significant impairment in GT compared to young mice [26], similar to the findings of Marmentini et al. [31]. However, other studies reveal no age-related changes in GT in male C57BL/6J mice [32,33]. Taken together, it appears the impact of aging on glucose homeostasis in the inbred C57BL/6J mouse is variable, perhaps due to comparing “aged” mice of different ages (16–18- vs. 21-27-month-old). Nonetheless, it is likely that GT improves in male C57BL/6J mice that are 21 months or older [26,33,34], and this improvement is likely due to beta cell hypertrophy and an increased insulin response to glucose [34]. These observations indicate that male C57BL/6J mice may not be an ideal pre-clinical model to study the impact of aging on glucose metabolism.

AMPK is a serine/threonine kinase that is thought to be a master regulator of cellular energy that controls glucose uptake, gluconeogenesis, and fatty acid oxidation [35]. In the present study, we acutely administered the AMPK activator, AICAR, and followed changes in blood glucose in Y, O, and VO mice. We demonstrate that VO mice are resistant to the AICAR’s ability to lower blood glucose. To the best of our knowledge, this is the first report to show that aged mice are resistant to the glucose lowering effect of AICAR, indicating impaired AMPK activation. However, a recent study demonstrated that AMPK expression and activity were lower in sarcopenic mice ranging in age from 23 to 30 months old [36]. In support of our findings, Reznick et al. [37] demonstrated that AMPK activity in response to an AICAR infusion was reduced in aged rodent muscle. However, no age-related differences were observed AMPK phosphorylation or isoform-specific activity in response to AICAR in the muscles of Fisher Brown Norway rats. Unfortunately, the present study was unable to attain quantifiable phospho-specific immuno-blots for AMPK, limiting our results to the in vivo data showing that acute AICAR treatment results in smaller changes in blood glucose in VO mice compared to Y and O mice. Unlike the VO mice, in the 18-month-old male C57BL/6J mice we did not observe a significant age-related decline in the blood glucose response to AICAR, indicating that a reduction in AICAR responsiveness, and perhaps AMPK activity, occurs later in life [33]. Because AMPK is a target of the anti-aging and anti-hyperglycemia drug metformin, it would be interesting to see how aging influences the response to metformin treatment. Although pre-clinical studies show benefits for metformin treatment when initiated before or at middle age, no study has examined the effectiveness of metformin when initiated in old age.

The present study has limitations that must be considered when interpreting the results. First, our results are contaminated by survivorship bias as the VO mice may be a select group that survived to 26–27 months because they were resistant to obesity and age-related declines such as lean body mass. However, we have followed C57BL/6J mice longitudinally from 20 months to 28 months old and demonstrated improvements in lean body mass, as well as declines in fat mass [26]. Although these longitudinal data downplay the survivor bias phenomena, we cannot be certain that the present findings are solely due to aging. Second, our results are limited by assessing lean body mass as a marker for sarcopenia but have not assessed muscle fiber size or strength. This limitation is marginalized to some extent because skeletal muscle is the largest component of lean body mass and we show a strong positive relationship between lean body mass and the skeletal muscle levels of AKT, a signaling molecule that plays a critical role in regulating muscle size [15]. Our study is also limited by our inability to attain quantifiable phospho-specific immuno-blots for AKT; therefore, we do not know if AKT signaling is enhanced in the VO mice. However, the increase in total AKT expression in the muscles of VO mice, in conjunction with the increase in lean body mass, suggests increased AKT signaling leading to greater protein synthesis and less protein degradation. Finally, studying only male mice limits our findings, although female C57BL/6 mice are less susceptible to age-related obesity and sarcopenia [26,36].

In summary, we demonstrate that VO (26–27 months old) male C57BL/6J mice are protected from age-related obesity and sarcopenia. The increased lean body mass in the VO mice is strongly related to the increase in AKT levels in skeletal muscle. The VO mice exhibited greater glucose tolerance and less fat mass than O mice (18 months old). Furthermore, glucose tolerance and body composition of Y (4 months old) and VO mice were quite similar. Interestingly, VO mice were resistant to the ability of the AMPK activator, AICAR, to lower blood glucose, indicating an impairment in AMPK signaling. Taken together, these findings provide important insight into the use of male C57BL/6J mice for aging studies designed to examine metabolism and sarcopenia, as the oldest of the old mice do not exhibit typical age-related declines.

## 4. Materials and Methods

### 4.1. Animals

Male C57BL6/J (Stock #000664) were purchased from Jackson Laboratories (Bar Harbor, ME, USA) at the age of eight weeks and were housed until the age of 4, 16–17, and 26–27 months old. The purchasing of the mice was staggered so that all mice were housed for at least three months and experiments were conducted with all age groups included. All mice were fed Prolab RMH 3000 normal chow. The mice were on a 12:12 h light–dark cycle with the lights turning on at 8:00 AM and off at 8:00 PM. The temperature and humidity of the animal housing room was approximately 23 °C and 40%, respectively. The current study followed the guidelines set forth by the National Research Council’s Guide for Care and Use of Laboratory Animals (Institute of Laboratory Animal Resources, Commission on Life Sciences, 2011), and the experimental protocol was approved by the Skidmore College Institutional Animal Care and use Committee (IACUC Protocol #143).

### 4.2. Body Composition

An LF50-BCA Minispec (Bruker Inc., Billerica, MA, USA) assessed the body composition of the mice. The Minispec is a nuclear magnetic resonance (NMR) system that allows for the quantification of fat mass and lean mass. NMR scans were completed by immobilizing fully conscious mice in a plastic tube that was placed in the instrument’s sample chamber for approximately 90 s. Body composition in mice assessed by NMR is highly correlated to the chemical analysis method of body composition assessment [20].

### 4.3. GT Testing

For glucose tolerance (GT) testing, the mice received an intraperitoneal injection of glucose (2.0 g/kg body weight) following an overnight fast (~15 h). Tail vein blood was collected (3–5 µL) at baseline and 20, 40, 60, and 90 min following the injection and blood glucose was measured using a hand-held glucometer (Accu-Check, Roche Diabetes Care, Inc., Basel, Switzerland) in duplicate, and averaged. The mice were allowed to recover for 7–10 days following the GT testing before they were subjected to insulin tolerance (IT) testing.

### 4.4. IT Testing

For IT tests, the same mice used for GT testing were injected with insulin (0.75 U/kg body weight) following a 6 h fast. Tail vein blood was collected (3–5 µL) at baseline and 20, 40, and 60 min following the injection and blood glucose was measured using a hand-held glucometer (Accu-Check, Roche Diabetes Care, Inc.) in duplicate, and averaged. The mice were allowed to recover for 7–10 days following the IT testing before they were subjected to N1-(β-D-Ribofuranosyl)-5-aminoimidazole-4-carboxamide (AICAR) tolerance (AT) testing.

### 4.5. AT Testing

For AT testing, the same mice used for the GT and IT testing were used and the procedure was identical to IT testing except mice were injected with AICAR (250 mg/kg), an activator of AMP-activated protein kinase (AMPK). Tail vein blood was collected (3–5 µL) at baseline and 20, 40, and 60 min following the injection and blood glucose was measured using a hand-held glucometer (Accu-Check, Roche Diabetes Care, Inc.) in duplicate, and averaged.

### 4.6. Energy Homeostasis

Energy expenditure was assessed using the OxyMax metabolic cage system (Columbus Instruments, Columbus, OH, USA). After 8 h to allow for adaptation to the metabolic cages, O_2_ consumption and CO_2_ production were measured for 24 h. During this time, the mice had free access to water and food. Energy expenditure during the light and dark cycles were calculated using the calorific value (3.815 + 1.232 × RER) × VO_2_ and expressed as kcals/day. Physical activity was assessed by an array of infrared emitters and detectors fitted to the OxyMax metabolic cage system. Caloric intake was assessed by giving the mice a known amount of chow for a given period of time (3–5 days). The remaining chow was weighed and subtracted from the initial amount of chow, multiplied by the kcal/g of the chow, and expressed as kcal/day.

### 4.7. Preparation of Muscle Extracts

The frozen quadriceps muscles were homogenized on ice using a motor-driven tissue grinder in RIPA Buffer (Cell Signaling Technology, Beverly, MA, USA) (1 mL buffer: 0.1 g muscle weight) containing protease and phosphatase inhibitor cocktails (Halt Protease/Halt Phosphatase, Thermo Fisher, Waltham, MA, USA). The homogenates were rotated at 4 °C for 30 min and then centrifuged at 9000× *g* for 30 min at 4 °C. The protein concentrations of the supernatants were determined by the BCA method (Pierce, Inc., Appleton, WI, USA). The remaining skeletal muscle extract was utilized for electrophoresis and immunoblotting experiments.

### 4.8. AKT Protein Expression

Skeletal muscle extracts and molecular weight standards (Bio-Rad, Hercules, CA, USA and Magic Mark, Invitrogen, Waltham, MA, USA) were subjected to SDS-PAGE. The proteins were then electrophoretically transferred to Immobilon membranes and immunoblotted with the AKT1 and AKT2 antibodies. After washing the membranes, the light generated by the horseradish peroxidase conjugated secondary antibody and Pico Plus chemiluminescence reagent (Thermo Fisher) was detected using a UVP digital imaging system (Analytik Jena, Upland, CA, USA). To account for gel loading differences, the immunoblots were stripped and re-probed with an α-tubulin antibody. The relative signal intensities of the AKT1, AKT2, and α-tubulin bands were determined by VisionWorks software (Version 8.2, Analytik Jena, Upland, CA, USA). All data were normalized to α-tubulin. All primary and secondary antibodies were purchased from Cell Signaling Technology (Beverly, MA, USA).

### 4.9. Statistics

All statistical analyses were carried out using commercially available software (GraphPad Prizm 9, San Diego, CA, USA). A repeated measures one-way analysis of variance (ANOVA) was used to compare the glucose values during the GT, IT, and AT tests among the age groups (Y, O, VO) over time (0–90 min). A one-way ANOVA was used to detect statistical differences in the area under the curve (AUC), body weight and body composition parameters, and AKT expression among the Y, O, and VO mice. For energy expenditure, a two-way ANOVA (age, and light phase) was utilized. Following a significant ANOVA main effect, a Tukey post hoc analysis was used to locate the significance. Pearson correlation was used to assess the strength of the relationship between AKT expression and lean body mass. Data are presented as mean ± SD, and the level of statistical significance was set at *p* < 0.05.

## 5. Conclusions

Since the C57BL/6J mouse is a widely used pre-clinical model to study aging, we sought to determine changes in body composition and in vivo glucose metabolism in the oldest of old male C57BL/6J mice. We observed an increase in lean body mass in VO mice compared to AG and Y mice, a finding that is explained, in part, by an increase in the expression of AKT protein levels in muscle. VO mice were also protected from obesity, an observation that is unrelated to changes in energy homeostasis. These findings provide important insight into the use of male C57BL/6J mice for aging studies designed to examine metabolism and sarcopenia.

## Figures and Tables

**Table 1:** The effect of advancing age on body composition in C57BL/6J male mice.

	Young (*n* = 7)	Old (*n* = 10)	Very Old (*n* = 9)	ANOVA *p* Value
Body Mass (g)	30.5 ± 1.28 *	47.8 ± 2.09 *	40.0 ± 1.82 *	0.0001
Fat Mass (g)	6.03 ± 1.16	23.8 ± 1.82 *	11.0 ± 2.09	0.0001
Lean mass (g)	20.1 ± 0.27	19.0 ± 0.63	23.1 ± 0.55 *	0.0001
% Body Fat	19.1 ± 2.90	49.2 ± 2.09 *	26.3 ± 3.75	0.0001

* Denotes statistically significant differences between all other groups by Tukey post hoc test, *p* < 0.015.

## Data Availability

The data are available upon reasonable request.
